# HFE protein impact on iron metabolism

**DOI:** 10.34763/devperiodmed.20172102.8590

**Published:** 2017-08-11

**Authors:** Barbara Kaczorowska-Hać, Jan Jacek Kaczor

**Affiliations:** 1 Zakład Terapii Zajęciowej Akademia Wychowania Fizycznego i Sportu w Gdańsku, Polska; 2 Katedra Fizjoterapii Akademia Wychowania Fizycznego i Sportu w Gdańsku, Polska

**Keywords:** mutacja HFE, żelazo, dzieci, HFE gene mutation, iron, children

## Abstract

Hereditary hemochromatosis type 1 is an autosomal recessive disorder caused by HFE gene mutations, which is an iron homeostasis metabolism controlling co-factor. Adults with male predomination present with clinical symptoms derived by iron overload in organs. The phenotype expression is individual with an influence of individual and environmental factors. Despite the fact that HFE variants are widespread, its impact still remains unknown. The article reviews the literature considering the role of HFE gene mutations regarding its impact in children.

## Streszczenie

Hemochromatoza typu 1 to schorzenie dziedziczone autosomalnie recesywnie, uwarunkowane mutacjami genu HFE, który jest współodpowiedzialny za kontrolę homeostazy metabolizmu żelaza. Objawy kliniczne, związane z przeładowaniem żelazem narządów wewnętrznych, takich jak wątroba, trzustka, serce, występują u osób dorosłych, głównie mężczyzn. Ekspresja fenotypowa choroby jest indywidualna, a na jej przebieg kliniczny ma wpływ szereg czynników osobniczych i środowiskowych. Znaczenie powszechnie występujących wariantów białka HFE w populacji wykracza poza klasyczną hemochromatozę narządową i dotychczas nie zostało w pełni wyjaśnione. Artykuł stanowi przegląd piśmiennictwa dotyczącego roli wariantów genu HFE w organizmie z uwzględnieniem aspektu gromadzenia żelaza w okresie rozwojowym.

## Wstęp

Żelazo jest niezbędnym mikroelementem dla życia organizmów: jest składnikiem hemu, białek uczestniczących w procesach energetycznych, metabolicznych oraz immunologicznych. Nieprzecenianą, funkcjonalną rolę tego pierwiastka umniejsza potencjalna toksyczność w reakcjach wolnorodnikowych [1, 2]. Stąd, metabolizm żelaza kontrolowany jest przez unikatowy system czynników i sensorów tak, by jego wchłanianie z przyjmowanym pokarmem nie przewyższało strat w stanie fizjologii, a aż 80 jego procent krążyło w organizmie w obiegu recyklingu. Gdy systemy kontroli homeostazy tego mikroelementu zawodzą, dochodzi do przeciążenia organizmu żelazem, co wzmaga stres oksydacyjny, indukuje reakcję zapalną oraz procesy włóknienia tkanek, i w konsekwencji, bez zastosowanego leczenia, nieodwracalnie uszkadza narządy wewnętrzne [3].

Hemochromatoza wrodzona (*ang. hereditary hemochromatosis, HH*) to choroba heterogenna, która obejmuje szereg uwarukowanych genetycznie zaburzeń metabolizmu żelaza, a konsekwencją ich jest przeładowanie organizmu tym pierwiastkiem. Zgodnie z klasyfikacją OMIM (On-line Medelian Inheritance in Man- http://www.ncbinim.nih.gov) wyróżniono cztery postaci wrodzonej hemochromatozy. Spośród nich najczęściej populacyjnie występuje hemochromatoza typu 1. U podłoża tego dziedziczonego autosomalnie recesywnego schorzenia leżą molekularne zaburzenia struktury genu *HFE* (*ang*. *Human Hemochromatosis Protein*). Kodowane przez ten gen białko HFE jest niezbędnym kofaktorem przemian regulujących homeostazę metabolizmu, pochodzącego ze spożytych produktów oraz z rozpadłych erytrocytów, żelaza. Niespecyficzne objawy kliniczne pod postacią zmęczenia, osłabienia i bólów stawów przepowiadają powolny, choć nieuchronny postęp choroby. Wieloletnie, nieleczone spichrzanie żelaza, powoduje szereg nieodwracalnych zmian w organizmie: niewydolność wątroby, kardiomiopatię, niedoczynność gruczołów wydzielania wewnętrznego, destrukcję stawów, czy hiperpigmentację skóry [3]. Spośród trzech najczęściej opisywanych mutacji genu hemochromatozy 1, dominujące znaczenie kliniczne ma substytucja cysteiny tyrozyną w pozycji 282 łańcucha białkowego (p.Cys282Tyr). Drugą najczestszą mutacją występującą u pacjentów HFE jest substytucja asparaginianu w pozycji 63 histydyną, a substytucja seryny cysteiną w miejscu 65 to mutacja Ser65Cys. Analizując ruchy migracyjne ludności, odnotowano geograficzny rozkład wymienionych defektów genetycznych [4]. Ma to niewątpliwie prehistoryczny związek z adaptacją genów określonych grup etnicznych do zmiennych klimatycznie warunków życia i dostępności pożywienia bogatego w żelazo, bądź wręcz przeciwnie, z redukcją ekspresji zmutowanego genu w okoliczności dostępu takiego pożywienia [5]. I tak, mutacja heterozygotyczna Cys282Tyr wykrywana jest u około 9% Europejczyków, a homozygotyczna u jednej na 200 do 400 osób, z przewagą rejonów północnych kontynentu. Natomiast wariant heterozygotyczny His63Asp występuje aż u 1/3 ludności hiszpańskiej, podczas gdy Ser65Cys spotykany jest sporadycznie [6]. Choć defekty molekularne genu *HFE* są populacyjnie powszechne, przeważnie w postaci heterozygotycznej, rzadziej homozygotycznej, a niekiedy mieszanej (heterozygoty mieszane), manifestacja kliniczna choroby obserwowana jest jedynie u niektórych probandów. Stwierdzono, że zaledwie 10-33% homozygot Cys282Tyr prezentuje objawy chorobowe, są to głównie mężczyźni w czwartej-piątej dekadzie życia [3]. Z kolei, spośród osób klinicznie demonstrujących chorobę, dominują homozygoty Cys282Tyr, stanowiąc aż 85-90 % pacjentów, a 3-5 % stanowią heterozygoty mieszane Cys282Tyr /His63Asp. Znaczenie alternatywnych wariantów His63Asp i Ser65Cys w postaci homozygotycznej, heterozygotycznej mieszanej, a tym bardziej heterozygotycznej, jak i heterozygotycznej Cys282Tyr jest nadal klinicznie niejasne [6, 7]. Jednak rola białka HFE dzięki swej strukturze i lokalizacji wykracza poza kanon regulacji metabolizmu żelaza.

## Homeostaza żelaza

Regulacja metabolizmu żelaza zachodzi już na etapie jego wchłaniania w enterocytach kosmków dwunastnicy, i podyktowana jest bieżącym zapotrzebowaniem organizmu. Zredukowane, pod wpływem enzymu ferroreduktazy dwunastniczej (DCytb1) kosmków, trójwartościowe jony żelaza niehemowego do postaci dwuwartościowej, transportowane są do cytozolu dzięki przezbłonowemu białkowemu przenośnikowi DMT1 (*ang*. *divalent metal transporter 1*). Hydrofobowe jony żelaza hemowego mają natomiast odrębny transporter - białko HCP1 (*ang. heme carrier protein 1*). W procesie eksportu żelaza z enterocytu do surowicy uczestniczą ferroreduktaza- hefajstyna (analog ceruloplazminy) i białko przezbłonowe ferroportyna [8, 9]. System reduktaz i oksygenaz komórkowych umożliwia nie tylko sprawną migrację jonów, która ma miejsce w samym cytozolu, również dzięki obecnemu w nim przenośnikowi DMT1, ale unieruchamia także pewną pulę żelaza w postaci rezerwuaru związanego z białkiem zapasowym - ferrytyną. Gdy aktualny stan metaboliczny organizmu nie wymaga nasilenia erytropoezy, żelazo zostaje usunięte wraz z ferrytyną złuszczających się fizjologicznie do światła przewodu pokarmowego enterocytów [10].

Równoległy, do zachodzącego w przewodzie pokarmowym, system kontroli, dotyczy odzyskiwania żelaza z rezerwuaru zużytych krwinek czerwonych, i jest de facto, głównym źródłem tego mikroelementu dla organizmu. Uwolnione, ze zdegradowanego przez enzym hemooksygenazę w fagosomach makrofagów układu siateczkowo-śródbłonkowego hemu, żelazo magazynowane jest w postaci ferrytyny, bądź eksportowane z komórki przez przezbłonową ferroportynę, a dalej transportowane surowicą przez białko transferynę do kolejnych komórek np. prekursorów erytrocytów w szpiku. Na powierzchni erytroblastów znajdują się dwa typy receptorów dla transferryny TfR1 oraz TfR2. Receptory TfR1 posiadają zdolność łączenia się z transferyną, i wraz z nią ulegają internalizacji do cytozolu. W komórce kompleks żelazo-transferyna-receptor TfR1 migruje dzięki DMT1, w endosomach ulega dysocjacji, by żelazo mogło zostać wykorzystane w zależności od potrzeb, np. w erytroblastach do syntezy hemu, a transferryna wraz z receptorem TfR1 powraca na powierzchnię komórki [11].

Regulacja zachowania homeostazy żelaza jest oparta o szereg mediatorów, które oddają aktualne zapotrzebowanie i stan metaboliczny organizmu. Stwierdzono, że kluczowym spośród nich jest produkowany w wątrobie 25-cio aminokwasowy peptyd hepcydyna, który powoduje degradację błonowego eksportera żelaza ferroportyny enterocytów i makrofagów. Ogranicza w ten sposób dostępność żelaza poprzez redukcję jego wchłaniania na poziomie przewodu pokarmowego jak i eksportu jonu uzyskanego na drodze recyklingu do surowicy [12].

Ekspresja genów kodujących kolejne elementy układu transportującego jony żelaza podlega regulacji licznymi czynnikami. W sytuacji niedoboru żelaza i niedotlenieniu komórki, modyfikujące białka żelaza IRP 1 i IRP2 (*ang. iron regulatory proteins*), łączą się z czynnikami IRE (*ang. iron regulatory elements*) mRNA stabilizując translację TfR1 i DMT1, blokując zaś ferrytyny i ferroportyny. Gdy w komórce występuje nadmiar żelaza, białka modyfikujące ulegają inaktywacji (IRP1) i degradacji (IRP2), co prowadzi do odblokowania translacji m-RNA ferrytyny i ferroportyny, natomiast synteza TfR i DMT1 ulega ograniczeniu. Z kolei, na poziomie transkrypcji TfR czynniki HIF 1α i HIF 1β (*ang. hipoxia inducible factors*) poprzez fragment promotora HRE (*ang. hipoxia responsive elements*) wzmacniają jego aktywność [10,13].

Ekspresja polipeptydu hepcydyny regulowana jest systemem sprzężenia zwrotnego opartego o układ związany z transmembranowymi receptorami transferryny 1 (TfR1) i transferyny 2 (TfR2). Posiadają one zdolność interakcji z obecnym również na powierzchni komórek białkiem HFE. Wprawdzie mechanizm współdziałania poszczególnych glikoprotein nie jest do końca wyjaśniony, jedna z hipotez zakłada, że gdy wzrasta wysycenie transferryny żelazem, kompleks TfR1 uwalnia białko HFE, które łącząc się z TfR2 tworzy kompleks, który działa jak sensor żelaza i uruchamia ekspresję genu hepcydyny [14]. Roli białka HFE upatruje się także we współzawodnictwie z transferryną o wiązanie z TfR1, co ogranicza stężenie wewnątrzkomórkowego żelaza.

Z kolei stymulacja ekspresji hepcydyny, w odpowiedzi na nadmiar żelaza, następuje w oparciu o aktywację cytoplazmatycznych białek przekaźnikowych z rodziny SMAD (nazwa utworzono z połączenia nazw białek homologicznych Sma oraz MAD wyizolowanych z organizmów *Caenorhabditis elegans* i *Drosophila melanogaster*), poprzez wiązanie jednego z białek morfogenetycznych kości BMP6 (*ang. bone morphogenetic protein*) do receptorów kinazy treoninowej w obecności koreceptora homojuveliny (HJV). Ufosforylowane białka SMAD1/5/8 tworzą w cytoplazmie kompleks z białkiem pośredniczącym SMAD4, którego celem po translokacji do jądra jest ekspresja genów kodujących hepcydynę [15]. Badania doświadczalne wskazują, że zarówno białko HFE jak i TfR2 posiadają funkcję modulatorową dla szlaku regulatorowego BMP6/SMAD. Białko HFE jest niezbędne do transportu i stabilizacji receptora dla BMP na powierzchni komórek jak i do prawidłowej interakcji BMP z receptorem [16, 17, 18].

Aktualny stan metaboliczny organizmu wymusza zmiany ekspresji hepcydyny, tak aby biodostępność żelaza była adekwatna do bieżącej sytuacji. W warunkach stanu zapalnego cytokina prozapalna IL-6 uruchamia fosforylację szlaku białek STAT (*ang. signals tranducers and activator of transcription*), głównie STAT3, które wzmacniają ekspresję genów, a co za tym idzie syntezę hepcydyny [19, 20, 21].

*Kolejnym regulatorem balansu żelaza jest opisane w 2014 r białko erytroferon uwalniane przez erytroblasty na skutek stymulacji erytropoetyną poprzez szlak Jak2/Stat5. Erytroferon działa supresyjnie na syntezę hepcydyny umożliwiając zwiększone przyswajanie żelaza niezbędnego do erytropoezy [22]*. I tak, w sytuacji niedotlenienia tkanek, czynniki HIF 1α, HIF 2 α i HIF 3 α przemieszczają się do jądra, gdzie z czynnikiem HIF 1β tworzą heterodimer, by wraz z cząsteczką HRE regulować transkrypcję erytropoetyny, która uruchamia syntezę erytroferonu.

Od czasu pierwszych klinicznych opisów hemochromatozy ustalono, jak wiele zawiłych szlaków przeplata się, by kontrola metabolizmu żelaza przebiegała skutecznie. Stwierdzono jednocześnie, że kluczowe mediatory tych procesów posiadają alternatywne, niekiedy nie do końca jasne, role.

## Funkcja białka HFE

Produktem, zlokalizowanego na krótkim ramieniu chromosomu 6 genu *HFE*, jest należące do rodziny MHC-1-like (*ang*. *major histocompatibility complex*) białko przezbłonowe HFE. Ta 343 aminokwasowa glikoproteina posiada w swej strukturze fragment transmembranowy, fragment cytoplazmatyczny oraz trzy domeny pozakomórkowe-α1, α2 oraz α3. Domeny α1 i α2 składają się z 8 łańcuchów β i dwóch helis α [23]. Białko o prawidłowej strukturze posiada pomiędzy domenami α2 i α3 mostek dwusiarczkowy niezbędny do niekowalencyjnego połączenia z β2 mikroglobuliną oraz do transportu z retikulum endoplazmatycznego na powierzchnię komórki, gdzie tworzy kompleks z TfR1 i TRf2 [24]. Interakcja białka HFE o prawidłowej strukturze i TfR jest kluczowa w mediacji ekspresji hepcydyny i modulacji gospodarki żelazowej. Produktami najczęściej występujących populacyjnie zmutowanych genów *HFE* są białka HFE Cys282Tyr i His63Asp. Ze względu na nieprawidłową budowę wariant Cys282Tyr białka HFE nie jest w stanie wiązać TfR, zaś wariant His63Asp ma ograniczone powinowactwo do TfR, co prowadzi do zaburzenia kontroli bilansu żelaza. U ok. 10-33% osób obciążonych zmutowanymi formami *HFE* dochodzi do to nadmiernego gromadzenia żelaza [25].

Jednakże, rola białka HFE wykracza poza regulację homeostazy żelaza, nie sposób bowiem pominąć funkcji jaką pełni w układzie immunologicznym organizmu. Wprawdzie podobne w swej strukturze do cząsteczek z klasy MHC Ia, wywiera jednak znacząco odmienną interakcję w rozpoznawaniu antygenu przez limfocyty T CD8+. W badaniach doświadczalnych wykazano, że prawidłowe białko HFE hamuje ich aktywację. Proces ten jest niezależny od interakcji z receptorem transferryny, ekspresji powierzchniowych HLA-A2, czy wiązania z β 2 mikroglobuliną. Białko HFE ponadto hamuje produkcję szeregu cytokin, chemokin, czy białek cytolitycznych (min. MIP- 1β, MIP-1α, INF-γ, IL-5, IL-8, IL-13). Obserwowana inhibicja procesów immunologicznych jest niezależna od rodzaju prezentowanego antygenu limfocytom T. Analizy doświadczalne wariantów białka HFE wykazują odmienną ich reakcję w modulacji supresji układu immunologicznego np. zmutowane białko Cys282Tyr nie blokuje prezentacji antygenów limfocytom T [26, 27].

## Polimorfizm genu *HFE*, a klinika

Proces kumulacji żelaza w organizmie ma przebieg powolny, a więc wieloletni, stąd zainteresowanie kliniczne hemochromatozą typu 1 dotyczy głównie populacji internistycznej. Jednakże ekspresja fenotypowa u obciążonej genetycznie osoby jak i rola czynników środowiskowych i epigenetycznych czyni jej przebieg osobniczo indywidualnym [28].

Wprawdzie nadmiar żelaza kumuluje się głównie w wątrobie, sercu oraz narządach wydzielania wewnętrznego, szczególnej uwagi wymaga analiza roli jonów żelaza, które poprzez generowanie reaktywnych form tlenu prowadzą między innymi do peroksydacji lipidów błon komórek, jak również tych wchodzących w skład cytozolu. Dotyczy to zwłaszcza tak wrażliwego na czynniki uszkadzające, centralnego układu nerwowego. Wbrew teorii o protekcji układu nerwowego przed kumulacją żelaza [29], istnieją liczne doniesienia o istotnej roli tego mikroelementu w rozwoju chorób neurodegeneracyjnych (choroby: Alzheimera, Parkinsona, Huntingtona oraz Stwardnienie Boczne Zanikowe; Amyotrhophic Lateral Sclerosis) [2, 30, 31]. Wprawdzie etiologia tych schorzeń jest złożona, w organizmach obciążonych mutacją genu *HFE*, jony żelaza poprzez uruchomienie kaskady reakcji wolnorodnikowych, upośledzoną homeostazę kwasu glutaminowego oraz zwiększoną fosforylację enzymu Pin 1 odpowiedzialnego za fosforylację amyloidu i białka tau, wpływają na procesy degeneracyjne komórek nerwowych [31,32, 33,34]. Istnieją również doniesienia o bezpośredniej roli, bądź modyfikacji czynników predysponujących do wystąpienia zawału niedokrwiennego mózgu [35, 36].

## Mutacje genu *HFE* u dzieci

Nie ulega wątpliwości, że żelazo jest niezbędne do wzrostu i rozwoju organizmu. I właśnie ta funkcja stanowi o podstawie jego roli. Nadmiar żelaza, a tym bardziej przeładowanie żelazem, to rzadko obserwowane, a zatem i opisywane zjawiska okresu rozwojowego [37]. Jednakże, w dostępnych, choć nielicznie prowadzonych, analizach pediatrycznych wykazano, że już dzieci obciążone mutacją genu *HFE* mogą wykazywać zaburzenia biochemiczne w zakresie jego gospodarki [38, 39]. Jednak, o ile wskazania do diagnostyki przyczyn podwyższonego stężenia żelaza u dorosłych są precyzyjne, świadomość potencjalnego zagrożenia objawową hemochromatozą pacjenta pediatrycznego w przyszłości wydaje się być nikła, a najczęściej wykonywane badania biochemiczne ograniczają się do izolowanego oznaczania stężenia żelaza w surowicy [38]. Populacja dziecięca, u której z określonych wskazań medycznych stwierdzono występowanie wariantów genu *HFE*, wydaje się być modelową grupą do obserwacji wpływu ewentualnych czynników środowiskowych na przebieg kliniczny choroby. Tak monitorowani pacjenci mają unikatową szansę na wczesne rozpoczęcie terapii i uniknięcie nieodwracalnych powikłań przeładowania żelazem. Już pacjenci w wieku rozwojowym, obciążeni mutacją genu *HFE*, demonstrują przekraczające normę wiekową wysycenie transferryny żelazem. Nie można zatem wykluczyć, że u dzieci obciążonych mutacją *HFE* zwiększona ilość żelaza może implikować określone zmiany biochemiczne i/lub patofizjologiczne, jak chociażby wpływ na nasilenie erytropoezy. Takie obserwacje poczyniono w grupie pediatrycznej obciążonej mutacją His63Asp, w której wykazano wyższe niż w zdrowej populacji [39] lub wręcz przekraczające normę stężenie hemoglobiny [40]. Zjawisko to wymaga pogłębienia badań, jakie szlaki sygnałowe i na drodze jakiego mechanizmu skutkują zjawiskiem intensyfikacji syntezy hemoglobiny w sytuacji zwiększonej dostępności żelaza. Z kolei, przy współistnieniu dodatkowych niekorzystnych czynników np. anemia hemolityczna, czy procedury medyczne obejmujące liczne transfuzje krwi, metabolizm żelaza może ulegać dalszym i głębszym zaburzeniom już u pacjentów w wieku rozwojowym obciążonym mutacją genu *HFE* [41,42].

## Podsumowanie

Analiza metabolizmu żelaza wymaga uwzględnienia złożonego systemu kontroli jego homeostazy. Ograniczeniem jest limitowanie zaburzeń biochemicznych oraz narządowych wynikających z nadmiaru tego mikroelementu wyłącznie do populacji dorosłej z marginalizowaniem ich wpływu na wiek rozwojowy. Wymiar struktury genu *HFE* daleko wykracza poza wyłączny standard koordynacji recyklingu żelaza. Powiązanie tych okoliczności daje obiecującą perspektywę dla kontynuacji badań nad złożona rolą genu *HFE*.
